# Case of Impacted Third Molar

**Published:** 1891-01

**Authors:** 


### Editorial.

### A Case of Impacted Third Molar.

November 1st, 1890, Mrs. P. presented herself for examination of an abscess, as she supposed, in the vicinity of the third superior molar of the right side.

The patient, thirty years of age; good health; good constitution. The mouth and teeth in a good condition except a fitulous opening on the outside of the gum just back of the second molar, about two lines above the margin of the gum. The mucous membrane of the entire mouth seemed very healthy, entirely up to the margin of the fistulous opening. There was no swelling; a mere trace of pus with a slight flow of serum.

The history of the case is about as follows: A little more than a year ago a slight enlargement occurred on the jaw opposite the normal position of the third molar. This was of slow growth, its greatest prominence not more than a line and a half, and its base diameter perhaps three lines. There was scarcely any soreness, and really no pain. After being in this condition about ten months it was examined by a dentist and by a physician; the latter suggested opening what was supposed to be an abscess cavity, and so, about two months ago an opening was made into it with an ordinary bistoury. There was little or no pus, but a small flow of what seemed to be serum. The cavity was washed out from time to time with various preparations, and during this time it became somewhat painful, though not seriously so. Some disturbance occurred about the roots of the second molar, though not enough at any time to prevent its use in mastication. A few weeks ago a small fungous seemed to spring from the orifice of the fistulous opening. This, however, was cut away and nothing of the kind has reappeared. As to the cause of the trouble neither the dentist or physician who examined it ventured an opinion. Though there had been but little pain or discomfort the patient and her husband manifested a good deal of anxiety about the case. In order, if possible, to ascertain the true condition an examination was made, and an impacted third molar was found situated about six lines above the margin of the orifice, with its crown leaning anteriorly against the posterior surface of the second molar. After ascertaining its position and knowing the healthy condition of the tissues round about, taking into account the health and vigor of the patient and the liability of injuring or displacing the second molar in an attempt to extract; it was decided not to remove the third molar, at least not till there should be indications of more serious trouble than has as yet appeared, and it was suggested to employ no treatment except such as may seem necessary to keep the parts entirely free from impurities. If any pus should be apparent, syringe gently with oxide of hydrogen ; and if the external opening is in the condition to exclude substances from the outside, and there is no more discharge than hitherto, simply let it rest. This is a case in which an immediate operation is not indicated, and probably taking all the conditions into account, never will be. If, however, it should prove a more serious offence, it can be at any time as well removed as now.

